# The Impact of Endurance Training on Human Skeletal Muscle Memory, Global Isoform Expression and Novel Transcripts

**DOI:** 10.1371/journal.pgen.1006294

**Published:** 2016-09-22

**Authors:** Maléne E Lindholm, Stefania Giacomello, Beata Werne Solnestam, Helene Fischer, Mikael Huss, Sanela Kjellqvist, Carl Johan Sundberg

**Affiliations:** 1 Department of Physiology and Pharmacology, Karolinska Institutet, Stockholm, Sweden; 2 Science for Life Laboratory, School of Biotechnology, Royal Institute of Technology (KTH), Solna, Sweden; 3 Department of Laboratory Medicine, Karolinska Institutet, Stockholm, Sweden; HudsonAlpha Institute for Biotechnology, UNITED STATES

## Abstract

Regularly performed endurance training has many beneficial effects on health and skeletal muscle function, and can be used to prevent and treat common diseases *e*.*g*. cardiovascular disease, type II diabetes and obesity. The molecular adaptation mechanisms regulating these effects are incompletely understood. To date, global transcriptome changes in skeletal muscles have been studied at the gene level only. Therefore, global isoform expression changes following exercise training in humans are unknown. Also, the effects of repeated interventions on transcriptional memory or training response have not been studied before. In this study, 23 individuals trained one leg for three months. Nine months later, 12 of the same subjects trained both legs in a second training period. Skeletal muscle biopsies were obtained from both legs before and after both training periods. RNA sequencing analysis of all 119 skeletal muscle biopsies showed that training altered the expression of 3,404 gene isoforms, mainly associated with oxidative ATP production. Fifty-four genes had isoforms that changed in opposite directions. Training altered expression of 34 novel transcripts, all with protein-coding potential. After nine months of detraining, no training-induced transcriptome differences were detected between the previously trained and untrained legs. Although there were several differences in the physiological and transcriptional responses to repeated training, no coherent evidence of an endurance training induced transcriptional skeletal muscle memory was found. This human lifestyle intervention induced differential expression of thousands of isoforms and several transcripts from unannotated regions of the genome. It is likely that the observed isoform expression changes reflect adaptational mechanisms and processes that provide the functional and health benefits of regular physical activity.

## Author Summary

Skeletal muscle is the most abundant tissue of the healthy human body. It is also highly adaptable to different environmental stimuli, *e*.*g*. regular exercise. Exercise training improves overall health and muscle function, and can be used to prevent and treat several common diseases *e*.*g*. cardiovascular disease and type II diabetes. Therefore, it is of great importance to understand the molecular mechanisms behind adaptation processes in human skeletal muscle. In this study, we show that different expression variants from the same gene can be regulated in different directions with training, implicating alternative protein functions from one single gene. Such findings are emblematic of the complex mechanisms regulating the effects of training. We also find that training changes the activity of functionally unknown parts of the genome, with the potential for new proteins involved in the health-enhancing effects of exercise. Additionally, our results challenge the belief of a skeletal muscle memory, where previous training can affect the response to a subsequent training period. Overall, we provide understanding of the skeletal muscle biology and novel insights into the mechanisms behind the massive benefits of regular exercise on the human skeletal muscle transcriptome, inspiring further studies for deeper investigation.

## Introduction

Long-term endurance training improves health and muscle function, as well as reduces mortality and risk for many diseases *e*.*g*. type II diabetes, obesity and cardiovascular disease [1]. How training induces these beneficial effects is not fully understood, but gene expression changes in skeletal muscle induced by training [2–4] are important aspects of the adaptation process and the ensuing health benefits. In addition to quantifying gene expression levels, RNA sequencing can determine global isoform expression levels and identify novel transcripts. These aspects have, to our knowledge, not been investigated in relation to human exercise training.

Alternative splicing greatly extends the complexity of the human transcriptome and, as a consequence, the proteome [5]. Almost 95% of all human genes have been shown to express different isoforms, and specific alternative splicing events are likely involved in for example determination of tissue specificity [6]. Specific splice variant expression has also been shown to be involved in adaptation to exercise training. For example, several isoforms of the transcriptional regulator PGC1α (Peroxisome proliferator-activated receptor gamma coactivator 1α), involved in mitochondrial biogenesis, are induced by exercise [7, 8]. Additionally, different splice variants of *VEGFA (*vascular endothelial growth factor A), important for increased vascularization, have been shown to increase to variable degrees in skeletal muscle in response to both resistance [9] and endurance exercise [10]. Furthermore, *IGF1* (Insulin Growth Factor 1) isoforms are differentially regulated by resistance exercise [11]. Although there is evidence for splicing event changes with exercise, there is no data available on the global isoform expression response to training.

Human skeletal muscle is a highly plastic tissue that readily adapts to different environmental stimuli. Regular resistance and endurance exercise training induces many physiological, structural, biochemical and transcriptional changes [12, 13]. If a standardized exercise training program is repeated, the changes are expected to be consistent within an individual, and largely, if not completely, lost upon training cessation [14]. Currently, there are no studies that have determined the consistency of global transcriptomic changes following repeated periods of training [15]. Previously, a study in mice has shown that resistance training could increase the number of myonuclei in skeletal muscle, an effect that was maintained after detraining [16]. In humans, use of androgenic anabolic steroids increases the number of myonuclei per myofiber that may persist long after periods of administration [17]. Such studies indicate that possible intrinsic ‘muscle memory’ mechanisms may be present after adaptations to physiological or pharmacological interventions. However, whether there is any skeletal muscle memory at the level of gene expression after endurance training has not been studied in humans. Additionally, there is a lack of studies investigating effects of repeated training in the same individual.

Accordingly, we aimed to study the impact of endurance training on the transcriptome, the consistency of the transcriptional response to repeated exercise training and the potential presence of skeletal muscle memory from previous endurance training. For this purpose, we adopted a one-legged training regime, where human volunteers trained only one leg during a first training period, with the other leg serving as an intraindividual control. One year after the start of the first period, a subset of the subjects started a second training period, this time training both legs. Skeletal muscle biopsies were collected before and after each training period to study the impact of repeated training, detraining and potential memory effects in the transcriptome, specifically regarding differential isoform expression and novel transcripts.

## Results

### Physiological, biochemical and transcriptional dynamics following repeated periods of endurance training

**Fig 1:**
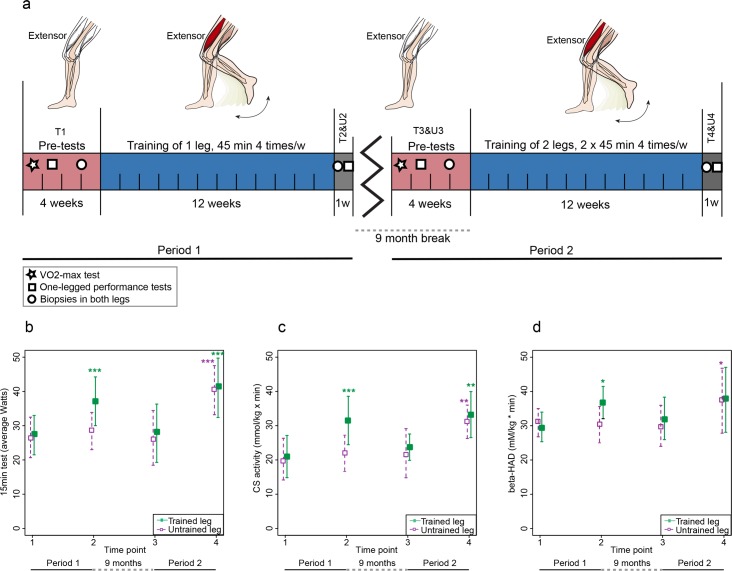
Experimental design and training response. **a)** The study was divided into two training periods (1 and 2) with a 9-month break in between. In Period 1, 23 individuals trained one randomized leg 45 min 4 times/week for 12 weeks, while the other leg remained untrained. In Period 2, 12 of the 23 individuals trained both legs with the same protocol as for the trained leg in Period 1, thus 2x45 min each session for 45 sessions in total. Both Periods included a 4-week “pre-test” phase in which individuals underwent a VO_2_-peak test, a one-legged performance test and biopsies were taken from both legs. After both training periods, skeletal muscle biopsies and performance tests were repeated for both legs; **b)** Performance results from the physiological 15-min test, **c)** Citrate synthase enzyme activity and **d)** β-HAD enzyme activity. Green squares represent the leg that trained in the Period 1, referred to as the trained leg, while purple squares represent the untrained leg, which was only trained in Period 2. Data is presented as mean ± SD for the 12 individuals that completed the whole study. Significance based on two-way repeated measures ANOVA is indicated by * (p<0.05), ** (p<0.001), *** (p<0.0001).

The influence of endurance training on physiological, biochemical and transcriptional properties of human skeletal muscle was studied in 23 volunteers [18]. All individuals underwent a 3-month endurance training period in which they exercised only one leg, with the other leg serving as an untrained control (Period 1) (Fig 1A). After a 9-month training break, twelve of the subjects underwent a second 3-month training period in which they exercised both legs, one at a time (Period 2). Before and after each training period, one-legged performance was evaluated and skeletal muscle biopsies were collected from both legs (Fig 1A, S1 Table). The samples were analyzed for enzyme activity of citrate synthase (CS) (a quantitative enzyme marker for the presence of intact mitochondria) and of β-HAD (3-hydroxyacyl-CoA dehydrogenase, involved in mitochondrial β-oxidation of fatty acids), both known to be consistently induced by endurance (aerobic) training [12]. From Period 1, we have previously published physiological and biochemical results, as well as transcriptome data at the gene level in the trained leg [18, 19]. Thus, transcriptome data for global splicing events, longitudinal data from the untrained leg in Period 1 and all data from Period 2 have not been published previously.

**Fig 2:**
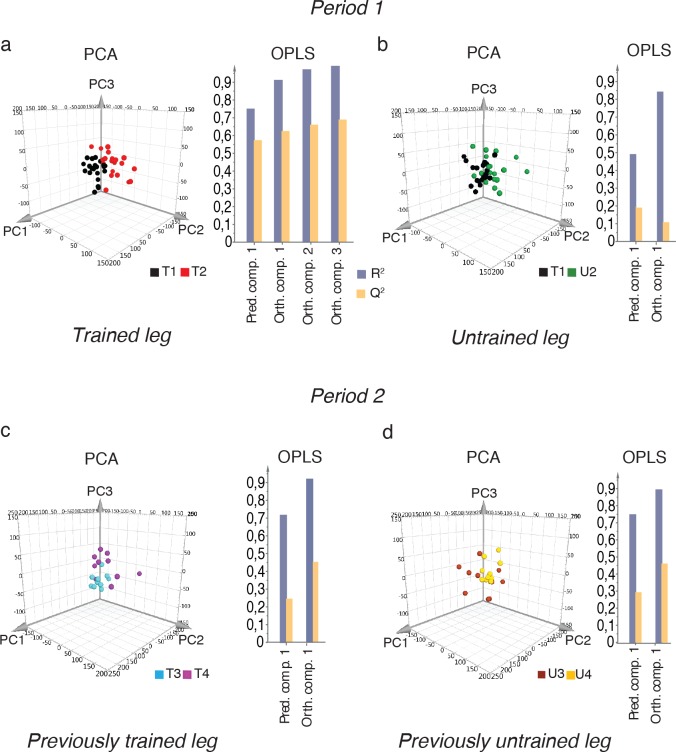
Global transcriptome changes with repeated periods of endurance training. Gene expression of 12,848 genes (mean FPKM>1) in total was analyzed using multivariate statistics. **a)** PCA (Principal Component Analysis) and OPLS (orthogonal projections of latent structures by means of partial least squares) of the training response in Period 1. The three-dimensional PCA score plot shows the PC1-3 plane, with biopsies collected before (T1, black) and after Period 1 in the trained leg (T2, red). The OPLS model shows the goodness of fit of the model, which represents the cumulative explained variance (R^2^, grey) and the goodness of prediction of the model, which represents the cumulative fraction of the total variance that can be predicted by the model from cross-validation (Q^2^, yellow), (n = 22 individuals). **b)** The specificity of the training effect was analyzed by comparing the untrained leg after (U2, green) to before (T1, black) Period 1, (n = 21). The training response in Period 2 in **c**) the previously trained leg (n = 10, before (T3) in blue and after (T4) in purple) and in **d**) the previously untrained leg (n = 11, before (U3) in dark red and after (U4) in yellow). For PCA and OPLS quality parameters, refer to Tables 1 and 2, respectively.

The one-legged knee extension endurance training resulted in marked physiological improvements (Fig 1B (mean±SD), S1 Fig (individual data)). These results were corroborated by the two biochemical parameters CS and β-HAD (Figs 1C and 1D, S2 and S3). To study transcriptome changes in response to training, RNA sequencing (RNA-seq) was performed on all skeletal muscle biopsies (S1 Table). Paired-end sequencing reads were aligned to the human genome and normalized gene expression levels were calculated as batch corrected FPKM (Fragments Per Kilobase of Exon per Million mapped fragments). Multivariate statistics, *i*.*e*. Principal Component Analysis (PCA) and orthogonal projections to latent structures by means of partial least squares (OPLS), were applied with a stringent significance level of 0.01 to analyze the global changes induced by the intervention. In Period 1, a clear response of the transcriptome was observed both at the gene (Fig 2A) and splicing event (S4 Fig, S2 and S3 Tables) levels for the trained leg. In PCA, the before-training (T1) and after-training (T2) samples formed two distinctive clusters in a PC score plot, and the OPLS model had high values for both the predictive and orthogonal components (Fig 2A, Tables 1 and 2). Moreover, to investigate if the training effect was specific only for the trained leg, we compared the before-training (T1) samples to the untrained leg after the first training period (U2). At the gene level, PCA was not able to distinguish two separate clusters and the predictive power of the OPLS model was small (Q^2^ = 0.20) (Fig 2B, Tables 1 and 2). Since these analyses, which were confirmed at the splice variant level as well (S4 Fig, S2 and S3 Tables), showed little differences, we concluded that the training effect was specific for the trained leg, as expected.

**Table 1:** Model quality parameters of PCA at gene level.

Model description	PCs	Number of samples	R_x_^2^	Q_x_^2^
T1vsT2	6	44	0.42	0.15
T3vsT4	4	20	0.50	0.10
U3vsU4	4	22	0.50	0.19
T2vsT3	4	24	0.46	0.17
T1vsT3	4	26	0.40	0.11
T1vsU3	3	24	0.41	0.16
U4vsT4	3	20	0.44	0.14
T3vsU3	4	24	0.50	0.20
T1vsU2	6	42	0.42	0.14

**Table 2:** Model quality parameters of OPLS at gene level.

Model description	PCs	Number of samples	R_x_^2^	R_y_^2^	Q _y_^2^
T1vsT2	1+3	44	0.31	0.99	0.69
T3vsT4	1+1	20	0.31	0.92	0.45
U3vsU4	1+1	22	0.28	0.88	0.46
T2vsT3	1+2	24	0.35	0.99	0.82
T1vsT3	1+1	26	0.18	0.91	0.15
T1vsU3	1+1	24	0.19	0.90	0.05
U4vsT4	1+1	20	0.27	0.93	-0.63
T3vsU3	1+1	24	0.27	0.80	-0.29
T1vsU2	1+1	42	0.18	0.85	0.12

During the nine months following the end of Period 1, the subjects were instructed to continue to live as they did before the study. Twelve of the subjects then started a second 3-month training period (Period 2). During Period 2, each leg was trained separately (*i*.*e*. one-legged training) and consecutively (with shifting order at each session) with the exact same workload protocol applied to the leg trained during Period 1 (Fig 1A). Period 2 induced similar physiological and biochemical changes to Period 1, but in both legs (Figs 1B–1D and S1–S3). At the gene level, PCA could partially distinguish two clusters for the biopsies collected before (T3) and after (T4) Period 2 in the previously trained leg. The predictive power (Q^2^) of the OPLS model was lower compared to Period 1, indicating the global transcriptome changes were not as evident (Fig 2C, Tables 1 and 2). Similar results were obtained for splicing events (S4 Fig, S2 and S3 Tables). A very similar OPLS model was also obtained for the previously untrained leg during Period 2 (U3 *vs* U4, Fig 2D).

We have previously shown that there are large sex differences in the human skeletal muscle transcriptome at baseline [19]. However, neither multivariate, nor univariate analyses were able to identify significant sex-specific transcriptional responses to training, although the baseline sex differences were similar in this study as well.

### Endurance training regulates multiple isoforms from the same gene in opposite directions

**Fig 3:**
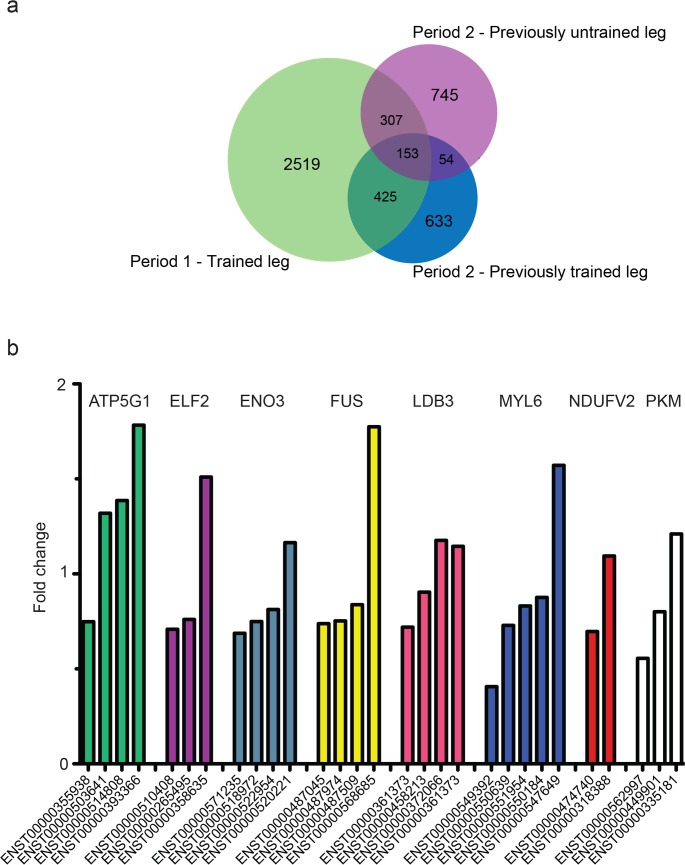
Training-induced differential isoform expression. **a)** Venn diagram representing the number of unique and shared differentially expressed isoforms from Period 1 (green) and both legs trained in Period 2 (blue for previously trained leg and purple for previously untrained leg). **b)** Examples of isoforms that were differentially expressed in different directions from the same gene in response in Period 1. The bars represent the mean fold change of each isoform (based on batch-corrected FPKM values), all are significant according to the OPLS model (S3 Table), (n = 22 individuals).

From the loading values of the OPLS models, we identified significant differentially expressed isoforms and genes. Over the first training period, 3,404 putative isoforms (S4 Table), belonging to 2,624 different genes, were differentially expressed (Fig 3A). Over 80% of these genes differentially expressed a single splice variant, while 13% and 4% expressed two and three splice variants, respectively. The remaining 2% of the genes differentially expressed between 4–8 different splice variants, the exception being *NDRG2*, where 17 variants decreased and one variant increased significantly with training. In total, 53% of the isoforms were downregulated with training, whereas 47% were upregulated.

Gene Ontology (GO) analysis showed that the differentially expressed isoforms were highly associated with *cellular respiration* (fold enrichment (FE) 5.1, false discovery rate (FDR) <10^−10^) and *ATP synthesis coupled electron transport* (FE 5.7, FDR < 10^−10^). Also at pathway level (using KEGG) *oxidative phosphorylation* (FE 3.8, FDR < 10^−10^) and the *TCA cycle* (FE 4.1, FDR < 0.001) were highly enriched. This is very similar to what has been observed for training-induced changes of gene expression at gene level [18].

Fifty-four genes had multiple differentially expressed isoforms that were also regulated in different directions with training. The mitochondrial ATP synthase *ATP5G1*, for example, had three protein-coding isoforms increasing in expression and one decreasing. Other interesting examples are also shown in Fig 3B. To identify the most training-responsive putative isoforms and validate the findings from Period 1, we compared those results with both legs trained in Period 2. Despite having a lower statistical power due to a smaller sample size in Period 2, 153 isoforms (belonging to 142 different genes) were differentially expressed in all trained legs (Fig 3A, S5 Table). GO analysis of these 153 isoforms resulted in a similar profile to the full isoform analysis, but with stronger enrichment. Specific genes of interest among the 153 included genes that also changed at gene level, *e*.*g*. the collagens (*COL4A1* and *COL4A2*), *HADHB* for lipid metabolism, three different isoforms of *PDHA1* for glucose metabolism and many genes involved in mitochondrial oxidative phosphorylation (*e*.*g*. *COX4I1*, *COX5A*, *NDUFA3*, *NDUFB5*, *SDHB* and *UQCRC2*).

All analyses conducted at isoform level were also performed at the gene level. For Period 1, we found 2,394 differentially expressed genes with similar enriched molecular functions and pathways to the isoform analysis. S5 Fig shows a Venn diagram comparing gene changes in the two periods. Differential gene expression at gene level from Period 1 has been reported previously [18].

### 34 novel transcripts regulated by exercise training

**Fig 4:**
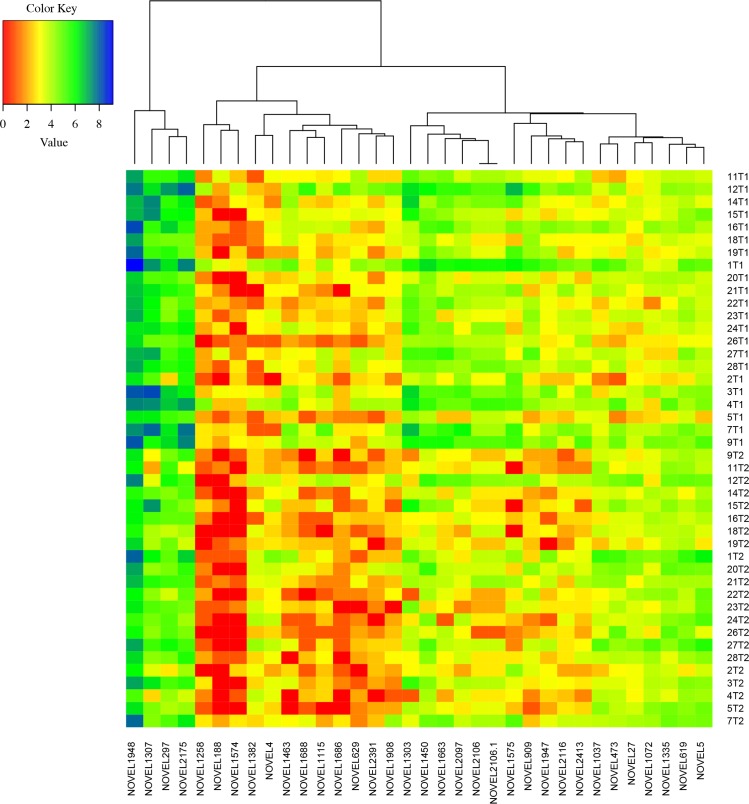
Differential novel transcript expression. Heatmap showing expression of differentially expressed novel transcripts in all individuals before (T1) and after (T2) training in Period 1. Read counts for each novel transcript per individual has been plotted as log_2_(read count), (n = 22 individuals).

RNA-seq analysis allows for discovery of novel transcripts and splicing events, and evidence from previous RNA-seq studies has demonstrated that the human genome is widely transcribed from both coding and non-coding regions [20]. To investigate whether exercise-induced expression changes occur also in unannotated parts of the genome, we used our recently published list of 2,430 novel transcripts expressed at baseline in human skeletal muscle [19]. Analysis of the trained leg from Period 1 using Limma [21] identified 35 novel transcripts (*i*.*e*. not included in Ensembl annotation v. 71) that were differentially expressed (Table 3, Fig 4). The novel transcripts were distributed across most chromosomes, and varied in size and expression level (Table 3). Two novel transcripts, NOVEL1037 and NOVEL1303, were identified as enhancer RNAs (eRNAs) based on comparison to a recent paper [22]. Further comparison to the RIKEN enhancer and TSS databases [22] confirmed that NOVEL1037 was enhancer-like, while NOVEL1303 had an H3K4me1 signal. Eight other novel transcripts were also defined as enhancer-like (S6 Table). We also investigated all transcripts using the Integrative Genomics Viewer (IGV) [23] and the UCSC Genome Browser [24]. One of the putative novel transcripts overlapped with a known gene, *PECAM1*, not present in the ENSEMBL annotation file but defined by RefSeq [25]. This gene is known to be induced by endurance training [26], which was hence confirmed by our results. Consequently, exercise altered the expression of 34 novel transcripts in total, nine were upregulated and 25 were downregulated. Two-thirds overlapped (partly or fully) with BodyMap skeletal muscle assembled transcripts and over 75% overlapped with conserved regions in chimpanzee, rhesus monkey, mouse, rat or dog according to UCSC Genome Browser. All 34 transcripts contained predicted open reading frames (ORFs) based on the ORF Finder Sequence Manipulation Suits (SMS) and between two and eleven protein-coding motifs (identified using Motif Search). The most common motif was the EGF-like domain signature 1 found in 30 out of 34 transcripts. This motif has an unclear functional significance, but is present in most animal proteins. Another common motif was 2Fe-2S ferrodoxin-type iron-sulfur binding region signature, found in 25 out of 34 transcripts. Ferrodoxins are small electron transfer proteins that are ubiquitous in biological redox systems, and the conserved region of the 2Fe-2S ferrodoxin family is found in various organisms including humans [27]. Additional information on the 34 novel transcripts is provided in Tables 3 and S6.

**Table 3:** Differentially expressed novel transcripts.

Novel transcript	Adj. p-value	Location(based on Hg19)	# of base pairs	logFC
NOVEL188	0.01	chr1:203,282,929–203,283,530	602	-1.57
NOVEL1463	0.01	chr3:50,706,681–50,708,607	1927	-1.22
NOVEL1335	0.01	chr21:42,651,004–42,654,447	3444	0.73
NOVEL629	0.01	chr13:111,514,476–111,515,829	1354	-1.24
NOVEL5	0.01	chr1:924,969–927,731	2763	0.68
NOVEL1688	0.02	chr5:10,330,080–10,332,584	2505	-1.35
NOVEL2175	0.02	chr8:79,528,923–79,538,540	9618	-1.03
NOVEL1072	0.02	chr2:49,456,766–49,457,039	274	0.71
NOVEL1948	0.02	chr6:141,724,706–141,729,312	4607	-0.58
NOVEL1686	0.02	chr5:10,314,797–10,316,503	1707	-1.42
NOVEL1382	0.02	chr22:28,070,090–28,070,354	265	1.20
NOVEL2106	0.02	chr7:123,311,643–123,315,063	3421	-0.92
NOVEL2413	0.02	chrX:137,696,914–137,699,450	2537	-0.88
NOVEL1663	0.02	chr4:186,419,228–186,420,365	1138	-1.16
NOVEL27	0.02	chr1:23,751,342–23,752,584	1243	0.65
NOVEL909	0.02	chr18:11,190,835–11,193,580	2746	-1.08
NOVEL473	0.03	chr12:32,148,185–32,151,464	3280	0.67
NOVEL1575	0.03	chr4:15,883,856–15,886,706	2851	-1.64
NOVEL4	0.03	chr1:917,725–918,046	322	0.82
NOVEL2106	0.03	chr7:123,311,643–123,315,063	3421	-0.99
NOVEL1450	0.03	chr3:46,555,187–46,556,521	1335	-0.61
NOVEL1307	0.04	chr20:61,192,866–61,198,737	5872	-1.53
NOVEL1258	0.04	chr20:1,399,556–1,400,476	921	-1.25
NOVEL1947	0.04	chr6:141,724,309–141,724,626	318	-0.72
NOVEL1037	0.04	chr2:27,961,139–27,962,389	1251	0.68
NOVEL619	0.04	chr13:100,737,624–100,738,981	1358	0.45
NOVEL1574	0.04	chr4:15,872,802–15,873,067	266	-1.38
NOVEL297	0.04	chr10:50,538,564–50,543,544	4981	-0.69
NOVEL2116	0.05	chr7:128,271,145–128,273,827	2683	-0.67
NOVEL1303	0.05	chr20:61,183,013–61,187,036	4024	-1.48
NOVEL1115	0.05	chr2:97479230–97480108	879	-1.00
NOVEL2391	0.05	chrX:75412145–75413589	1445	-0.95
NOVEL2097	0.05	chr7:113,279,670–113,283,698	4029	-0.91
NOVEL1908	0.05	chr6:99,312,552–99,314,806	2255	-0.85

Five novel transcripts were validated using quantitative real-time PCR. The mean expression changes agreed with the RNA-seq results in all cases, with two transcripts showing statistical significance, while there was a trend for the remaining three (p≤0.1) (S6 Fig). S6 Fig also shows qPCR validation of the expression of two genes at gene level (IGF2 and FABP4) and two specific isoforms (of MUSTN1 and SPARC), that all significantly changed in the same direction with training in Period 1 as measured by RNA-seq.

In Period 1, 61 previously unannotated putative splice variants of known genes were also found to change with training (S7 Table). Examples included *A2M*, *FABP3*, *KRT222*, *NOS2* and *TACO1*.

### Lack of evidence for an endurance training-induced skeletal muscle global transcriptome memory

**Fig 5:**
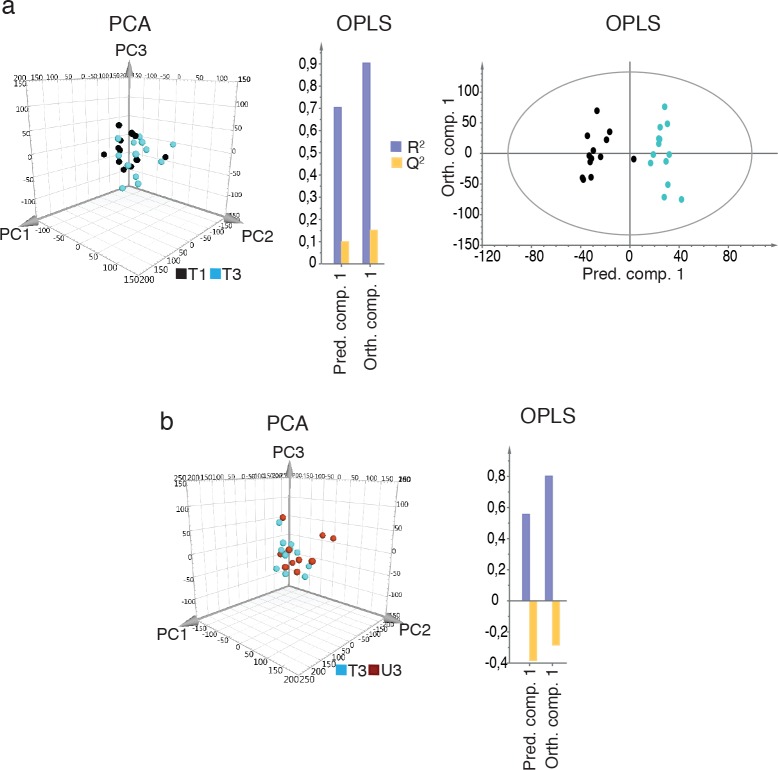
Endurance-induced skeletal muscle memory at the transcriptome level. Human skeletal muscle gene expression data (12,848 genes) was used to study: **a)** The presence of any residual effect in the previously trained leg by comparing before training in Period 1 (T1, black) with the same leg before Period 2 (T3, blue). Left: results are presented as a 3D PCA score plot showing the PC1-3 plane. Middle: summary of fit of OPLS; R^2^ (grey): goodness of fit of the model, which represents the cumulative explained variance; Q^2^ (yellow): goodness of prediction of the model, which represents the cumulative fraction of the total variance that can be predicted by the model from cross-validation. Right: 2D score plot of OPLS (n = 13) **b)** Transcriptome differences between the previously trained leg (T3, blue) and the previously untrained leg (U3, brown) before Period 2. Left: a 3D PCA score plot showing the PC1-3 plane. Right: summary of fit of OPLS; R^2^ and Q^2^ as described above (n = 12). For PCA and OPLS quality parameters, refer to Tables 1 and 2, respectively.

One purpose of repeated training of a previously trained leg compared to a previously untrained leg was to investigate the possible presence of skeletal muscle memory represented at the transcriptome level. To investigate if any transcriptional effects remained from the first training period, we conducted two different analyses. First, we compared samples before Period 1 (T1) with the previously trained leg before Period 2 (T3). PCA could not separate the samples clearly, but the OPLS model showed a small difference between these two time points (predictive power Q^2^ of 0.15) (Fig 5A, Tables 1 and 2) indicating small transcriptome differences. To investigate if the differences were an effect of previous training of the trained leg (*i*.*e*. muscle memory) or simply the time between sample collections (one year between samples preceding Periods 1 and 2), we compared biopsies from the trained leg before Period 2 (T3) with the untrained leg at the same time point (U3). We assumed any effect of time to be the same on both legs. PCA could not separate the sample groups, and the OPLS model clearly showed no significant difference between the biopsies (the predictive power of the model was negative) (Fig 5B, Tables 1 and 2). Moreover, there were no specific genes that differed significantly between the two legs before Period 2. Therefore, these results show that the first training period did not lead to any detectable remaining effects at the overall transcriptome level after a 9-month break. However, the rate of perceived exertion (Borg-scale) in the exercising leg from the end of the first training session in Period 2 was lower (perceived as easier) in the previously trained leg (mean±SD T-leg: 13.3±2.6, U-leg 14.0±2.9, p = 0.05).

Additionally, we examined whether transcription could be affected to a different degree following repeated training in the “primed” trained leg in comparison to the previously untrained leg. For this purpose we compared the effect of training on differential gene expression in the different legs following period 2 (T3 *vs* T4 and U3 *vs* U4). As expected, training induced significant gene expression changes in both legs. In the previously trained leg, 852 genes significantly changed (T3 *vs* T4), while in the previously untrained leg, 928 genes changed (U3 *vs* U4). Interestingly, only 180 of those genes were common between the two legs. However, when comparing the two legs after Period 2 (T4 *vs* U4), the biopsies could not be separated in the PCA and the OPLS showed no statistical difference between the two groups (Tables 1 and 2, S2 and S3).

### Physiological, biochemical and transcriptional alterations in response to repeated endurance training

**Fig 6:**
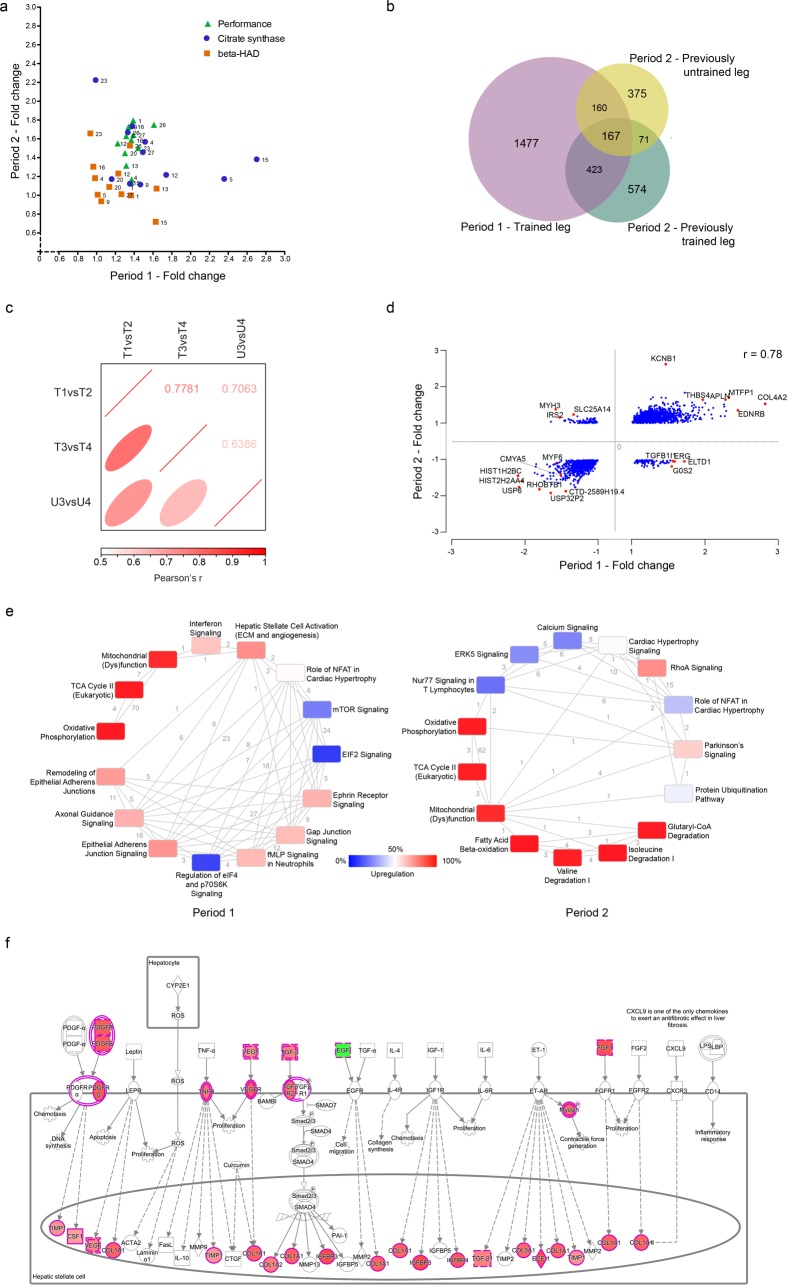
Comparing intra-individual effects of a repeated environmental training stimulus. **a)** Correlation of individual fold changes for performance (green triangles), citrate synthase activity (blue circles) and β-HAD activity (orange squares) for the leg trained in Period 1 (T1 *vs* T2) and the same leg trained in Period 2 (T3 *vs* T4) for the 12 individuals that completed both training periods. **b)** Venn diagram comparing the gene changes in the leg trained in Period 1 with both legs trained in Period 2. Data is based on all available sequencing data from biopsies of the common 12 individuals. **c)** Pearson’s correlation between the absolute fold changes of the significant genes differentially expressed in Period 1 (T1 *vs* T2) with the absolute fold changes of the same genes in Period 2, for T3 *vs* T4 and U3 *vs* U4, respectively. All correlations were significant (p<0.0001) **d)** XY-plot of the fold changes of the same genes as in (c) for the leg trained in Period 1 (T1 *vs* T2) and the same leg trained in Period 2 (T3 *vs* T4). Examples of genes are highlighted in red with gene names. **e)** Ontology analysis of the differentially expressed genes in Period 1 (left, T1 *vs* T2) and the same leg in Period 2 (right, T3 *vs* T4) for 12 individuals. Top 15 categories based on significance according to IPA are shown. The number at each line represents the number of common genes between the two categories connected by the line. Square color indicates the percentage of upregulated genes among the significant differentially expressed within that category. **f)** Example of a network that was uniquely enriched in Period 1 (T1 *vs* T2). Red color indicates an increased expression of that gene or group of genes with training, while green color indicates a decrease.

There is a lack of knowledge of how the same individual responds to repeated biological interventions, including training. Given this, we sought to determine if an individual responds the same when exposed to the same environmental stimulus a second time. To investigate this we performed a specific analysis comparing the response to Period 1 and 2 only for the 12 individuals that took part in both training periods. Physiological performance, β-HAD and CS activity unexpectedly correlated rather poorly between Period 1 and Period 2 (Fig 6A). OPLS analysis of the gene changes in the 12 individuals showed that Period 1 induced greater and more robust transcriptome changes compared to Period 2 (S8 Table). A Venn diagram of the gene changes in all three comparisons is shown in Fig 6B. Gene ontology analysis of the genes that changed significantly in at least two comparisons showed a clear enrichment of functional categories highly relevant for training adaptation, including *cellular respiration* (FE 10, FDR<2*10^−26^) and the Kegg pathway *oxidative phosphorylation* (FE 8.4, FDR<7*10^−37^), categories that did not appear as significant when analyzing genes changing uniquely in one comparison. To further investigate the repeated training response, we compared the fold changes of the 2,227 differentially expressed genes in Period 1 with the fold changes in Period 2 (T3 *vs* T4) (Fig 6C and 6D). Over 90% of the 2,227 significant genes in Period 1 had an absolute fold change in the same direction in T3 *vs* T4, *i*.*e*. repeated training in the same leg one year later. When including the same genes for the previously untrained leg as well (U3 *vs* U4), almost 80% changed in the same direction in all three comparisons. Consequently, the correlation for gene changes in response to repeated training was high (Fig 6C). Despite this, there were also specific and highly interesting differences in the responses. Based on Ingenuity pathway analysis (IPA), Periods 1 and 2 showed similar changes for oxidative phosphorylation but different for ECM (extracellular matrix) reorganization and angiogenesis (Fig 6E and 6F). All significantly enriched canonical pathways are listed in S9 Table (T1 *vs* T2) and S10 Table (T3 *vs* T4). IPA also identifies upstream regulatory factors that could be driving the observed alterations in gene expression. As expected, Period 1 and 2 had several transcriptional regulators in common, *e*.*g*. ATF6, TP53 and the cytokine SPP1. Uniquely represented in Period 1 were, for example, ERG and SPDEF, members of the ETS family of transcription factors, which have been shown to be enriched in regions with a decrease in DNA methylation with endurance training in Period 1 [18]. AMPK and the estrogen receptor are two additional examples that appeared uniquely in Period 1 (see S11 Table for significant upstream regulators). Unique to Period 2 were for example PPARGC1B, known to be involved in regulating mitochondrial biogenesis in response to training [28], and ESRRA (see S12 Table for significant upstream regulators for Period 2, T3 *vs* T4).

## Discussion

We applied RNA sequencing to a total of 119 human skeletal muscle biopsies, to thoroughly investigate the repeated training-induced transcriptome at both the gene and isoform levels. The RNA-seq technology is becoming a standard method to quantify RNA expression [29]. To our knowledge, this dataset represents one of the very few large human skeletal muscle RNA-seq data collections currently available and the only one with repeated sampling following an environmental lifestyle intervention. Multivariate statistics were used to analyze this large dataset to account for interdependences among genes [30]. Also, multivariate data analysis techniques have already been successfully applied to gene/isoform expression analyses [19, 31, 32].

The one-legged training regime in this study has the advantage of including an intraindividual control leg (the untrained leg). The only difference between the two legs was the training stimulus, since both legs had the same genome and were exposed to identical environmental factors (*e*.*g*. diet, stress and sleep) and the same systemic arterial blood composition. This unique control allowed us to isolate training-specific effects on the transcriptome. The control leg was, however, not completely unaffected by training since exercise-related changes in blood flow and an increased concentration of hormones and growth factors are able to induce effects outside the exercising leg [33]. The exercising muscle itself secretes myokines into the circulation [34], with potential endocrine effects.

More than 3,000 isoforms were significantly changed with training in Period 1. Many of the specific genes and their splicing events are highly biologically interesting. The SPARC gene had five different isoforms that significantly increased between 1.5- and 3-fold. Suppression of SPARC (or osteonectin) in skeletal muscle of mice causes myofiber atrophy [35] and it is also expressed at lower levels in beta cells of type II diabetic patients [36]. An isoform of *TGFB1I1* (or HIC-5) was induced >3-fold with training. It is a coactivator of the androgen receptor and its multiple splice variants have been shown to be involved in myogenesis [37]. Three isoforms of the *MUSTN1* gene were downregulated by 1.1–1.7 times. MUSTN1 (Musculoskeletal, Embryonic Nuclear Protein) has been shown to be markedly upregulated following one bout of resistance exercise [38]. Of interest among the 153 isoforms that changed in both periods was a decrease in one splice variant of the HIPK3 protein kinase. This gene has not been reported in association with exercise training before. Its protein phosphorylates the transcriptional regulators c-Jun and RUNX2 [39]. *JUN* is activated by skeletal muscle contraction in rats [40] and the RUNX2 related family member RUNX1 has been predicted as an important transcriptional regulator of the training-induced transcriptome in humans [2].

In this study, 54 genes had isoforms that changed in different directions, which indicates potential alternative functions in training adaptation and highlights a problem with measuring gene expression only at gene level. It could also be due to a change in the cellular composition of the muscle with training, as different splicing events are commonly associated to specific cell types and tissues [6]. The most broadly regulated isoform set was *NDRG2*, a member of the N-myc downregulated gene family, which belongs to the alpha/beta hydrolase superfamily. In C2C12 myotubes it has been identified as a PGC1α/ERRα transcriptional target, which influences protein turnover and the regulation of genes involved in muscle contraction and function [41]. NDRG2 is phosphorylated in response to insulin stimulation and has been identified as a mediator of the cardioprotective effects of insulin on ischemia-reperfusion injuries following myocardial infarctions [42]. Seventeen of the 18 differentially regulated isoforms of *NDRG2* (NDRG Family Member 2) were downregulated between 1.3 and 2.9 times. ENST00000397855 was the only NDRG2 splice variant that was upregulated (FC 2.2). A human resistance training study showed that *NDRG2* gene expression was downregulated during hypertrophic conditions [43]. The *FUS* (Fused in Sarcoma) gene encodes an RNA-binding protein involved in pre-mRNA processing, including splicing [44]. Three FUS isoforms were downregulated with training, all retained introns according to Ensembl, while one protein-coding isoform was upregulated (FC 1.8). Overexpression of *FUS* leads to motor neuron loss and muscle atrophy associated to development of ALS (Amyotrophic lateral sclerosis) [44, 45], while physiological upregulation of *FUS* could have implications for alternative splicing regulation. *PKM* (Pyruvate kinase, Muscle) encodes an ATP-producing glycolytic enzyme and had three protein-coding isoforms differentially expressed with training, two decreased and one increased significantly. In relation to exercise training, alternative isoforms of PGC1α (*PPARGC1A*) have been proposed to have different functions based on the observations that they are induced to a different degree following different forms of exercise training [7, 8]. No splice variants of PGC1α were significantly induced by training in the present study. However, this was not unexpected as most splice variants have been shown to go back to baseline levels 24h after an acute bout of exercise [46], and the biopsies in the present studies were obtained at that time point.

A total of 34 novel transcripts changed in response to three months of endurance training. All contained open reading frames and predicted protein-coding motifs, indicating protein-coding potential. Two transcripts were identified as enhancer transcripts (eRNAs) when compared to a recent study by Arner *et al*. 2015 [22]. eRNAs have been suggested to support interaction between the enhancer and the genes it regulates [47]. eRNA transcription is typically very low, in accordance with our novel eRNA transcripts. Also, a change in enhancer transcription has been linked to changes in gene activity of nearby genes [48]. Although functional analyses are required to fully confirm that the novel transcripts are relevant to exercise training responses, these results are interesting as a first indication of how transcription from unannotated regions may be important for exercise training adaptation.

There is a notion in the popular science literature that once a person has been fit it is easier to become fit again. This belief is commonly attributed to a skeletal muscle memory, despite a lack of scientific evidence. After nine months of detraining there were no significant remaining effects in one-legged performance with regard to skeletal muscle enzymatic markers or the global transcriptome. However, there might be limitations to this analysis, *i*.*e*. batch effects (despite applying batch correction) and lack of power in Period 2, which could have blunted possible effects. Furthermore, there could be other remaining effects, *e*.*g*. differences in fiber type distribution, vascularization or epigenetic patterns. Strength training in mice and anabolic steroid use in humans have been shown to increase the number of nuclei in skeletal muscle cells, which persists after cessation of training and drug intake, respectively [16, 17]. In relation to endurance training, one study has shown that a 20% increase in CS activity with training was maintained after eight weeks of detraining [49], however the majority of the changes are presumed to be gradually lost upon training cessation [14].

Despite not finding any significant evidence of an endurance-induced skeletal muscle transcriptome-level memory after detraining with this experimental set-up, a molecular memory mechanism could exist at *e*.*g* the epigenetic level and potentially induce a different response in the previously trained leg compared to the previously untrained leg. Apart from a significant increase of β-HAD activity in the previously untrained leg only, there were no significant physiological differences in response between the two legs. At the transcriptome level, the second training period induced significant changes in both legs. Although there were differences in the specific gene changes between the two legs, there was a clear fold change correlation between the genes that changed in Period 1 and those that changed in both legs trained in Period 2. The overall ontology was also similar between the two training periods. This shows that the transcriptome response to training was rather consistent, although differences in the specific genes affected and the degree of physiological and biochemical change exist. Interestingly, the smallest correlation was observed between the gene changes of the two legs trained in Period 2. The rate of perceived exertion during the first session in Period 2 was significantly lower in the previously trained leg, and although this measurement is inherently impossible to blind, both observations could implicate some remaining effects from previous training. Furthermore, the reduction in the number of gene changes in Period 2 compared to Period 1 could be attributed to some unknown, possibly systemic, memory effect invoked by training of the previously trained leg at the start of Period 2. Because the reduction was observed in both legs, the effect would have to be systemic, affecting both legs, rather than local to the previously trained leg. Comparing the trained leg in Period 1 to the same leg retrained in Period 2, over 90% of genes numerically changed in the same direction, while 168 genes did not. Even though none of these 168 significantly changed in Period 2, this could reflect a possible memory mechanism associated to these genes. The data is therefore not convincing enough to conclude the presence of a training-induced skeletal muscle memory, but not exclude it either.

Whether individuals, physiologically or transcriptionally, respond consistently to repeated exercise regimens is very poorly addressed [15]. Any such within-subject variability in training response can only be evaluated with experiments that repeatedly expose an individual to a standardized exercise regimen. The design of the present study allowed us to address this. It was clear that repeated exercise periods led to quite consistent changes of a sub-set of genes, but also that a large fraction of individual genes changed significantly only in one training period. Such inconsistency may be due to true but random within-subject variability or differences in diet or other lifestyle domains. It may also be due to methodological and power issues with some genes reaching significance in one training period but fail to do so in the other. One category that appeared significant only in Period 1 was hepatic stellate cell activation. The included genes, as illustrated in Fig 6F, were related to extracellular matrix remodeling (*e*.*g*. the collagens) and angiogenesis (*e*.*g*. *VEGF*, *PDGF* and TGF-beta1), both functionally important also in skeletal muscle adaptation to training [50, 51].

In conclusion, endurance training altered over three thousand human skeletal muscle isoforms, highly associated to oxidative ATP production. Interestingly, several isoforms expressed from the same gene were regulated in opposite directions, which may indicate different functions. That 34 novel transcripts, all with protein-coding potential, were regulated provides a first indication that transcriptional regulation of hitherto unannotated regions might have relevance for exercise training adaptation. We found no coherent evidence of a skeletal muscle transcriptome memory, even though there were some data indicating a training-induced memory mechanism. These findings show the great need for highly controlled studies of repeated exercise training to provide further insights into intra-individual biological response variability and the potential presence of skeletal muscle memory.

## Materials and Methods

### Study participants

Before the study, named EpiTrain, the experimental protocol was explained to all subjects, and written informed consent was obtained. The study was approved by the Ethics Committee of Karolinska Institutet (approval number: 2008/1747-31/2) and conformed to the Declaration of Helsinki. Twenty-three young, healthy, non-smoking, volunteers (eleven females and twelve males), participated in the study. Baseline characteristics, including fitness level based on a VO_2_ peak test (mean ± SD 39.0 ± 3.8 ml*kg^-1^*min^-1^), have been published previously [18].

### Experimental design

Before training, one-legged performance was assessed through a 15-minute optimal performance test, as previously reported [18]. Skeletal muscle biopsies were obtained under local anesthesia by the percutaneous needle technique [52] from the *vastus lateralis* muscle of both legs and immediately frozen in liquid nitrogen. All subjects subsequently started the first training period, training one randomized leg four times per week for 12 weeks. All participants completed a total of 45 training sessions. For more details on the training protocol, see Lindholm *et al*. [18]. Twenty-four hours after the last training session of the first training period, skeletal muscle biopsies were collected again from both legs and the performance test was repeated within one week after the last session (Period 1).

Nine months later, twelve of the subjects (six males and six females, mean ± SD age 28.5 ± 3.8 years, height 173.8 ± 8.6 cm, BMI 25.9 ± 4.0 kg*m^-2^ and VO_2_ peak 40.3 ± 4.3 ml*kg^-1^*min^-1^) continued to perform a second training period (Period 2). The same one-legged performance test was conducted and biopsies were obtained from both legs before and after the training. Period 2 consisted of exactly the same training as the first period (same workload protocol and number of sessions), but now for both legs, one at a time with shifting order at each session. The workload exerted on each leg was thus the same as for the one leg trained in Period 1, but the whole-body energy expenditure was double in Period 2. This repeated training period has not been reported previously. The full experimental design is illustrated in Fig 1A.

### Enzyme activity assays

Enzyme activity was measured in the skeletal muscle biopsies from all time points and legs. The procedure has been previously described [18]. In brief, freeze-dried biopsies were homogenized in 0.1 M phosphate buffer (pH 7.7) with 0.5% BSA. For citrate synthase (CS), the tissue lysates were mixed with a reagent solution (0.1 M Tris-HCl, 2.5 mM EDTA, 0.5 mM L-malate, 512.5 nM NAD^+^, 399μg MDH). Acetyl-CoA started the reaction and the velocity was registered with a fluorometer. β-HAD activity was measured in a reagent solution (0,5M Imidazol (pH 7), 0.1M EDTA, 5mM NADH diluted in carbonate buffer, 2mM AcAcetyl-CoA) where addition of the tissue lysate started the reaction. Fluorecence of β-HAD oxidation of NADH to NAD^+^ was recorded and related to a standard curve.

### RNA extraction, quality control and sequencing

Total RNA was extracted by the Trizol method (Invitrogen, Carlsbad, CA, USA) according to the specifications from the manufacturer. The concentration and quality of the total RNA was determined using the RNA 6000 Nano chip on the 2100 Bioanalyzer automated electrophoresis system (Agilent Technologies Inc., Santa Clara, CA, USA). Two μg of total RNA was used for poly-A-selection followed by library preparation of each sample, which was subsequently bar-coded and prepared (Illumina, San Diego, CA, USA). The libraries were clustered on a cBot cluster-generation system using an Illumina HiSeq paired-end cluster-generation kit and sequenced as paired-end, 2x100 bp on the Illumina HiSeq 2000. All lanes were spiked with 1% phiX control library. All 119 samples underwent sequencing.

### Sequence read alignment and analysis

2.43 billion paired-end reads underwent quality and adapter trimming using Trim Galore v. 0.2.7 (www.bioinformatics.babraham.ac.uk/projects/trim_galore/). Subsequently, the processed reads were aligned to the human genome reference hg19 with TopHat version 2.0.4 [53] using standard parameters except for:—solexa1.3-quals -p 8 –no-discordant as well as–transcriptome-index used on an index built from ENSEMBL v71 transcript annotation. Transcript assembly of the aligned reads was performed by using Cufflinks version 2.1.1 [54] with the max-bundle-frags option set to 1e9 and Fragments Per Kilobase of Exon per Million mapped fragments (FPKM) values were calculated using the same software. The aligned reads and the software HTSeq version 0.5.1 [55] were used to count the number of reads per gene. The gene counts generated by HTSeq were used for differentially expressed analysis using Limma [21]. Cufflinks gene and isoform FPKM values, which were first corrected for batch effect using ComBat [56], were used for differential expression analysis in Simca p+13.0.3x64 (Umetrics). Differential expression of previously unannotated splicing events was performed using Ballgown [57].

### Multivariate data analysis

The identification of differentially expressed genes and isoforms, using the software Simca p+13.0.3x64 (Umetrics), was conducted applying the multivariate statistical tools Principal Component Analysis (PCA) and Orthogonal Projections to Latent Structures by means of partial least squares (OPLS). The software was run with default parameters except for “Confidence level on parameters” that was set to 99% and “Significance level” that was set to 0.01. The application of multivariate data analysis techniques to gene/isoform expression analyses has already been described [31, 32]. Briefly, OPLS calculates the systematic variation in **X** matrix and divides it into two parts: one that is linearly related to **Y** (and therefore predictive) and one that is unrelated (orthogonal) to **Y** [56, 58]. Data pre-processing for PCA and OPLS analysis, both for gene and isoform expression data (mean FPKM >1), was based on mean-centering and unit variance scaling. Cross-validation was applied to estimate the model complexity [59]. The analyses were performed comparing leg biopsies collected before and after the first training period. Model parameters for PCA are shown in Tables 1 and S2, whereas for OPLS in Tables 2 and S3. 12,848 genes and 23,418 isoforms were included in the different time point comparisons.

OPLS model loading values, together with their jack knife confidence levels, were used to calculate the significance of differentially expressed isoforms/gene [58, 59]. More precisely, the significance was calculated as absolute value, ABS (loading)–ABS (jack knife confidence interval). Genes and isoforms with a positive value of significance indicated that a gene/isoform was significant.

### Gene ontology and network analysis

For functional and pathway annotation of all differentially expressed isoforms and genes as well as a subset of the differentially expressed isoforms, the DAVID functional annotation tool (http://david.abcc.ncifcrf.gov) was used. Differential expression was also analyzed with the Ingenuity pathway analysis software (Ingenuity Systems, Inc., Redwood, CA, USA; www.ingenuity.com) with standard parameters and including the “Causal network” option. In the Ingenuity software the isoform IDs were automatically converted to the corresponding gene IDs by the software itself.

### Novel transcript discovery and analysis

The 2,430 novel human skeletal muscle transcripts published by Lindholm *et al*. [19] were subject to a differential expression analysis using the Bioconductor package Limma [21]. By comparing the biopsies for the trained leg taken before and after the first training period we identified in total 34 differentially expressed novel transcripts not included in Ensembl annotation v. 71 and not defined by RefSeq [25]. The transcripts were compared with BodyMap skeletal muscle assembly (GEO accession GSE30611). Conserved regions overlapping with chimp, rhesus, mouse, rat and dog were analyzed using UCSC Genome Browser [24]. Open reading frames were defined using ORF Finder Sequence Manipulation Suits (SMS) and a subset of the genes were analyzed using Motif Search (www.genome.jp/tools/motif).

### qPCR validation

Two known genes, two isoforms and five novel transcripts were validated using quantitative real-time PCR. cDNA was synthesized using 2 μg of total RNA and the High Capacity cDNA reverse transcription kit (Applied Biosystems, Carlsbad, CA, USA) in a total volume of 20 μl (in 23 individuals before and after training for the trained leg). Taqman reagents were used for known genes and RPS18 was used as an endogenous control (FABP4 Hs 00609791-m1, IGF2 Hs 00171254.m1, RPS18 Hs01375212-m1, Applied Biosystems, Carlsbad, CA, USA). For isoforms and novel transcripts, primers were designed to identify MUSTN1 ENST00000513520 (forward: GCACCTTGGAAGCACCAATA, reverse: AGCAGTGAAAGCAGGACGAG), and SPARC ENST00000521569 (forward: CCTGTCTCTAAACCCCTCCA, reverse: TCCTCTGCACCATCATCAAA), Novel 629 (forward: GCCCTTGGTGTAGCTTGTGA, reverse: GAAGCCGACCAATGAAAAGGG), Novel 1072 (forward: GTTAGGCCACCCACAGTGAA, reverse: GGAATGGACGTAGACACACG), Novel 297 (forward: CAAGTGATCCACCCACATTG, reverse: AGGGGTCATCAGCTTCCATT), Novel 1450 (forward: CAGAATGGTCTTTGGGTGAAA, reverse: GGGTCGTTAGAGCAGACGTT) and Novel 1663 (GGCAATTGGCAAGCTTTATC, reverse: CCAATGGGGAGACTGATTGA) with RPS18 (forward: CTTCCACAGGAGGCCTACAC, reverse: CCATCGATGTTGGTGTTGAG) as an endogenous control. The reaction volume was 10 or 15 μL, including 1–4 μL of sample cDNA diluted 1:25, primers and Taqman Fast Universal PCR Master Mix or SYBR Green PCR Master Mix (Applied Biosystems, Carlsbad, CA, USA). All SYBR Green quantification reactions were verified with a melting curve and size of transcript was verified on an agarose gel.

### Data availability

Data are available on GEO under the accession numbers GSE58608, GSE60590 and GSE60833.

## Supporting Information

S1 Fig Individual performance data. Individual measurements of the physiological one-legged 15-min performance test for both legs at the different time points of the study. The trained leg, in blue, refers to the leg that underwent training in both periods, while the untrained leg, in red, refers to the leg that was trained only in Period 2. Data is shown for the 12 individuals that finalized both periods of the study.
(PDF)

S2 Fig Individual citrate synthase activity data. Individual measurements of the enzyme activity of citrate synthase for both legs at the different time points of the study. The trained leg, in blue, refers to the leg that underwent training in both periods, while the untrained leg, in red, refers to the leg that was trained only in Period 2. Data (in mmol/kg*min) is shown for the 12 individuals that finalized both periods of the study.
(PDF)

S3 Fig Individual β-HAD activity data. Individual measurements of the enzyme activity of β-HAD for both legs at the different time points of the study. The trained leg, in blue, refers to the leg that underwent training in both periods, while the untrained leg, in red, refers to the leg that was trained only in Period 2. Data is shown for the 12 individuals that finalized the whole study.
(PDF)

S4 Fig Multivariate analysis of human skeletal muscle isoform expression data. Based on 23,418 isoforms. From a to f): three-dimensional PCA score plot showing the PC1-3 plane. **a)** before (T1, black) and after (T2, red) training in Period 1, **b)** before (T1, black), and after (U2, yellow) Period 1 for the untrained leg, **c)** before (T3, blue) and after (T4, purple) training in Period 2 of the leg trained in Period 1, **d)** before Period 1 (T1, blue) and before Period 2 (T3, black), **e)** before Period 2 of the previously trained leg (T3, blue) and previously untrained leg (U3, dark red), and **f)** before Period 1 (T1, black) and before Period 2 of the previously untrained leg (U3, dark red).
(PDF)

S5 Fig Venn diagram of the gene changes in Periods 1 and 2. Data is based on loadings from the OPLS analysis shown in Fig 2.
(PDF)

S6 Fig Quantitative real-time PCR validation. Differentially expressed; known genes **a)** FABP4 and **b)** IGF2, known isoforms **c)** MUSTN1 (ENST00000513520) and **d)** SPARC (ENST00000521569) (n = 23) and novel transcripts **e)** Novel 629, **f)** Novel 1072, **g)** Novel 297, **h)** Novel 1450 and **i)** Novel 1663 before and after training in Period 1 (n = 20). RPS18 was used as an endogenous control for all transcripts. Data is presented as mean ± SD. * p<0.05, ** p<0.01.
(PDF)

S1 Table All skeletal muscle biopsies collected. Samples marked in red failed RNA sequencing quality control and were excluded from transcriptome analysis.
(XLS)

S2 Table Model quality parameters of PCA at isoform level. (XLS)

S3 Table Model quality parameters of OPLS at isoform level. (XLS)

S4 Table Differentially expressed isoforms with training in Period 1. (XLS)

S5 Table 153 isoforms differentially expressed in Period 1 and both legs trained in Period 2. (XLS)

S6 Table Extended information on the 34 differentially expressed novel transcripts. (XLS)

S7 Table Differentially expressed novel isoforms. (XLS)

S8 Table Model quality parameters of OPLS at gene level for all available samples in 12 individuals. (XLS)

S9 Table Significantly enriched canonical pathways in Period 1 (T1 *vs* T2) from Ingenuity pathway analysis. (XLS)

S10 Table Significantly enriched canonical pathways in Period 2 (T3 *vs* T4) from Ingenuity pathway analysis. (XLS)

S11 Table Significant predicted upstream regulators in Period 1 (T1 *vs* T2) from Ingenuity pathway analysis. (XLS)

S12 Table Significant predicted upstream regulators in Period 2 (T3 *vs* T4) from Ingenuity pathway analysis. (XLS)
