# Supplementary material for: The Impact of Endurance Training on Human Skeletal Muscle Memory, Global Isoform Expression and Novel Transcripts
Source: PLoS Genet. 2016 Sep 22;12(9):e1006294. doi: 10.1371/journal.pgen.1006294 (PMC5033478; doi:10.1371/journal.pgen.1006294)
Supplement: S1 Fig — Individual measurements of the physiological one-legged 15-min performance test for both legs at the different time points of the study. The trained leg, in blue, refers to the leg that underwent training in both periods, while the untrained leg, in red, refers to the leg that was trained only in Period 2. Data is shown for the 12 individuals that finalized both periods of the study. (PDF) [file pgen.1006294.s001.pdf]

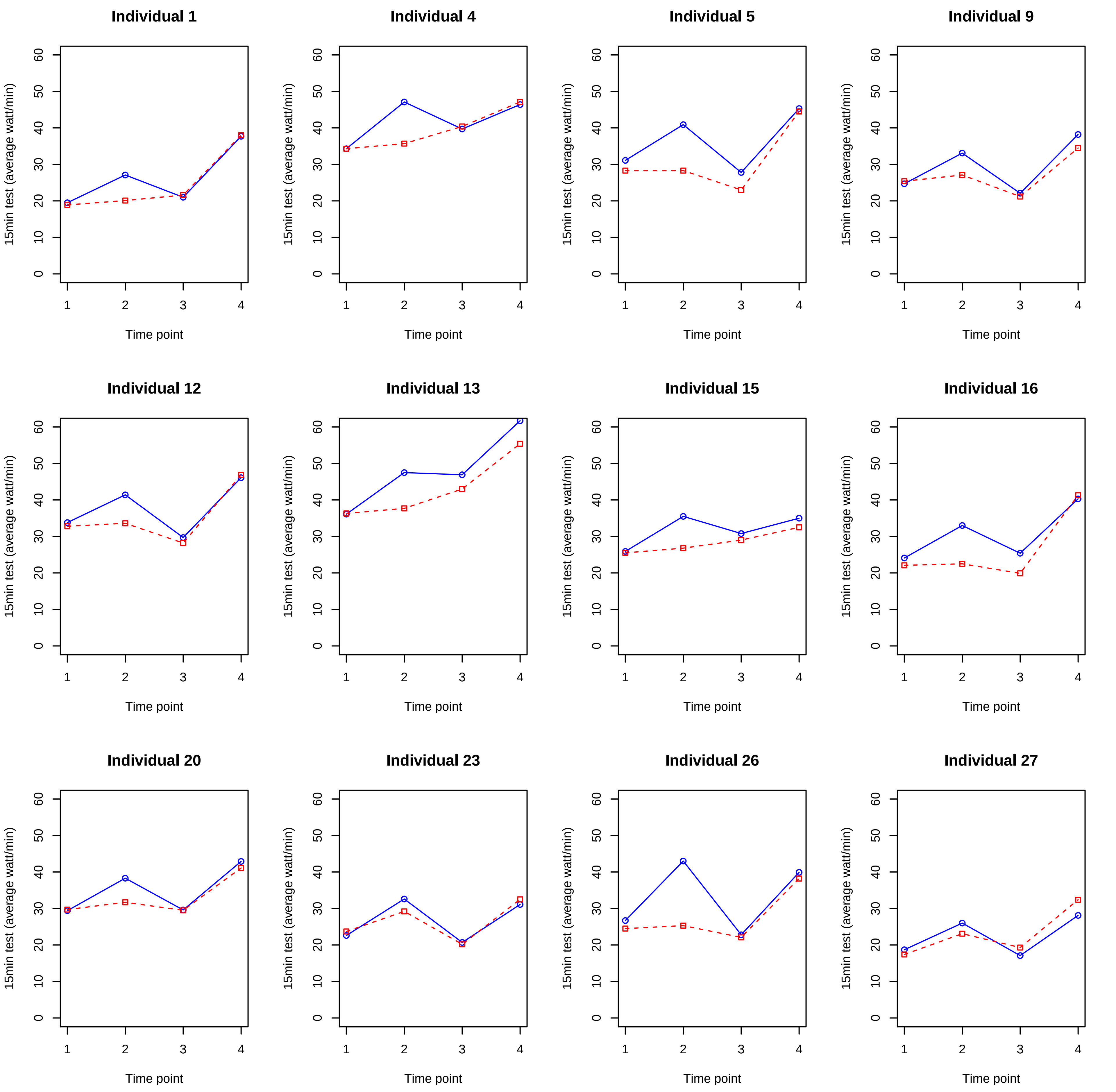
Individual 1
Individual 4
Individual 5
Individual 9
Individual 12
Individual 13
Individual 15
Individual 16
Individual 20
Individual 23
Individual 26
Individual 27
15min test (average watt/min)
0
10
20
30
40
50
60
Time point
1
2
3
4
