# Supplementary material for: The Impact of Endurance Training on Human Skeletal Muscle Memory, Global Isoform Expression and Novel Transcripts
Source: PLoS Genet. 2016 Sep 22;12(9):e1006294. doi: 10.1371/journal.pgen.1006294 (PMC5033478; doi:10.1371/journal.pgen.1006294)
Supplement: S2 Fig — Individual measurements of the enzyme activity of citrate synthase for both legs at the different time points of the study. The trained leg, in blue, refers to the leg that underwent training in both periods, while the untrained leg, in red, refers to the leg that was trained only in Period 2. Data (in mmol/kg*min) is shown for the 12 individuals that finalized both periods of the study. (PDF) [file pgen.1006294.s002.pdf]

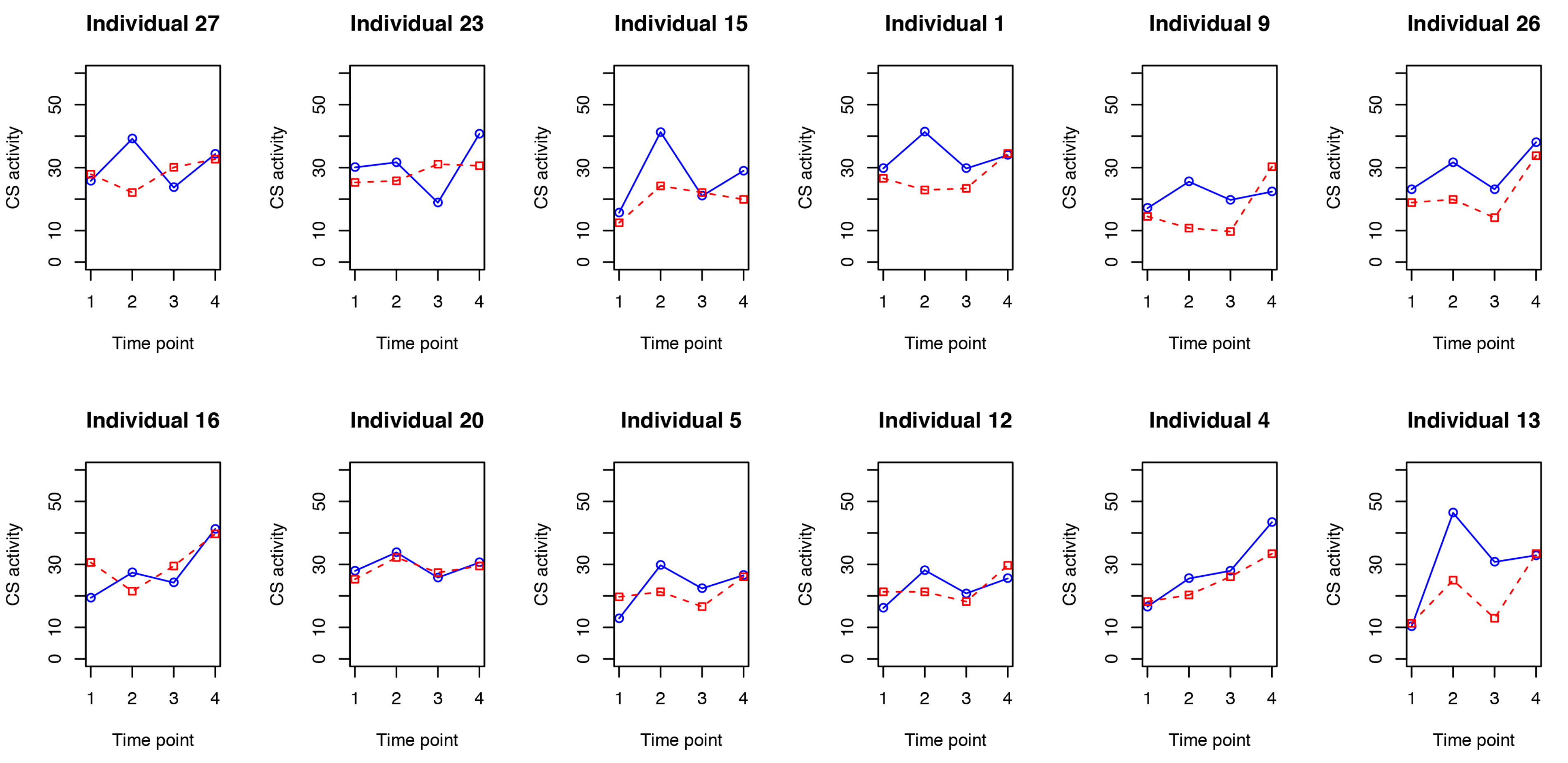
Individual 27
Individual 23
Individual 15
Individual 1
Individual 9
Individual 26
Individual 16
Individual 20
Individual 5
Individual 12
Individual 4
Individual 13
CS activity
Time point
0
10
30
50
1
2
3
4
