# Supplementary figures and images for: The Impact of Endurance Training on Human Skeletal Muscle Memory, Global Isoform Expression and Novel Transcripts
Source: PLoS Genet. 2016 Sep 22;12(9):e1006294. doi: 10.1371/journal.pgen.1006294 (PMC5033478; doi:10.1371/journal.pgen.1006294)

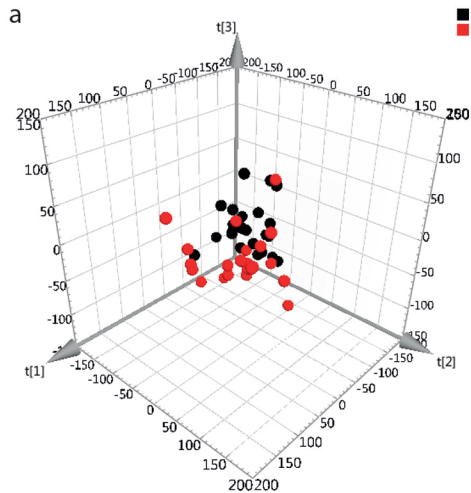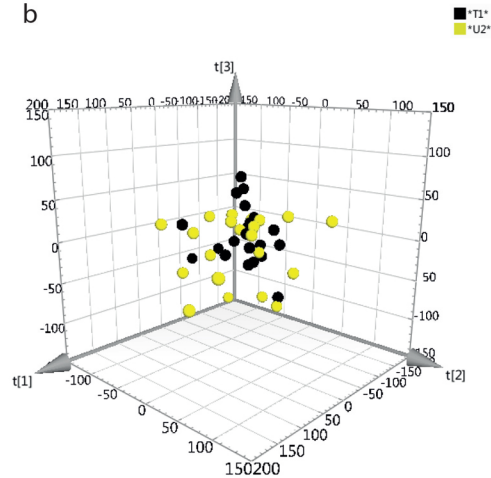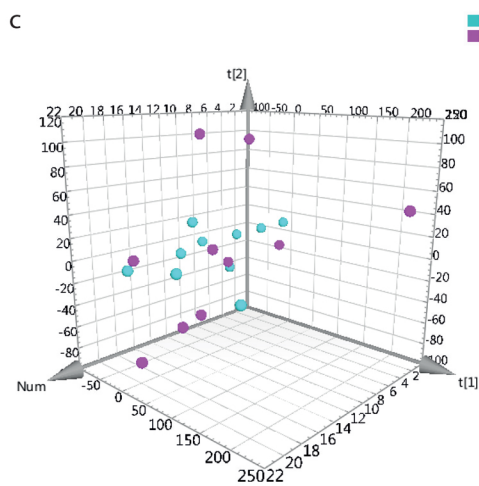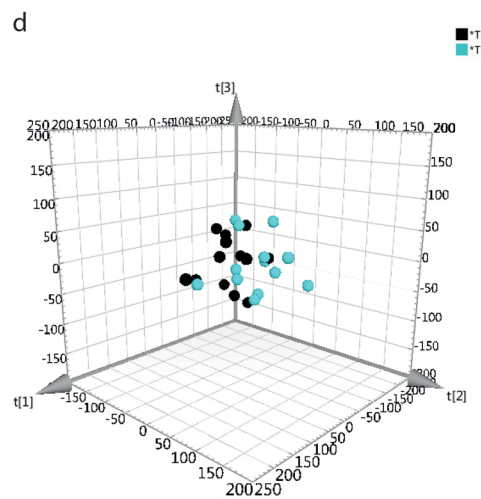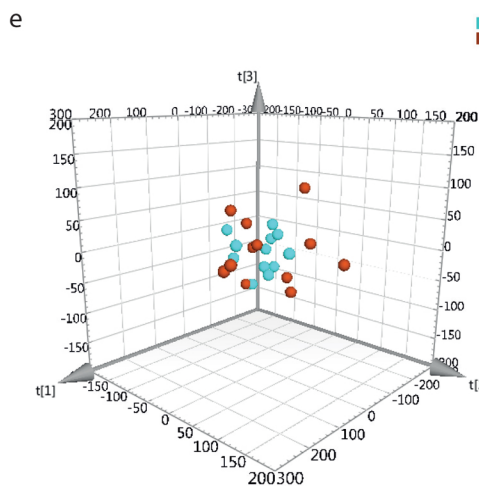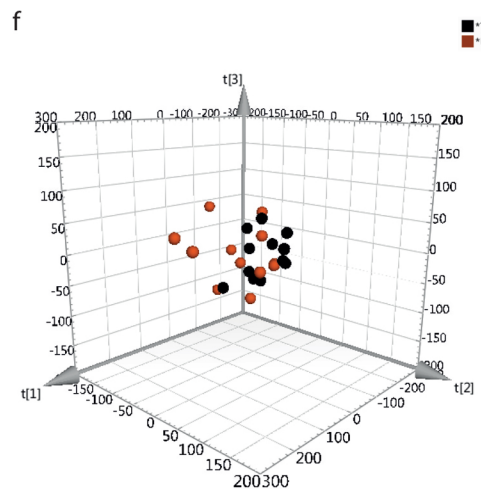

Supplement: S4 Fig — Based on 23,418 isoforms. From a to f): three-dimensional PCA score plot showing the PC1-3 plane. a) before (T1, black) and after (T2, red) training in Period 1, b) before (T1, black), and after (U2, yellow) Period 1 for the untrained leg, c) before (T3, blue) and after (T4, purple) training in Period 2 of the leg trained in Period 1, d) before Period 1 (T1, blue) and before Period 2 (T3, black), e) before Period 2 of the previously trained leg (T3, blue) and previously untrained leg (U3, dark red), and f) before Period 1 (T1, black) and before Period 2 of the previously untrained leg (U3, dark red). (PDF) [file pgen.1006294.s004.pdf]

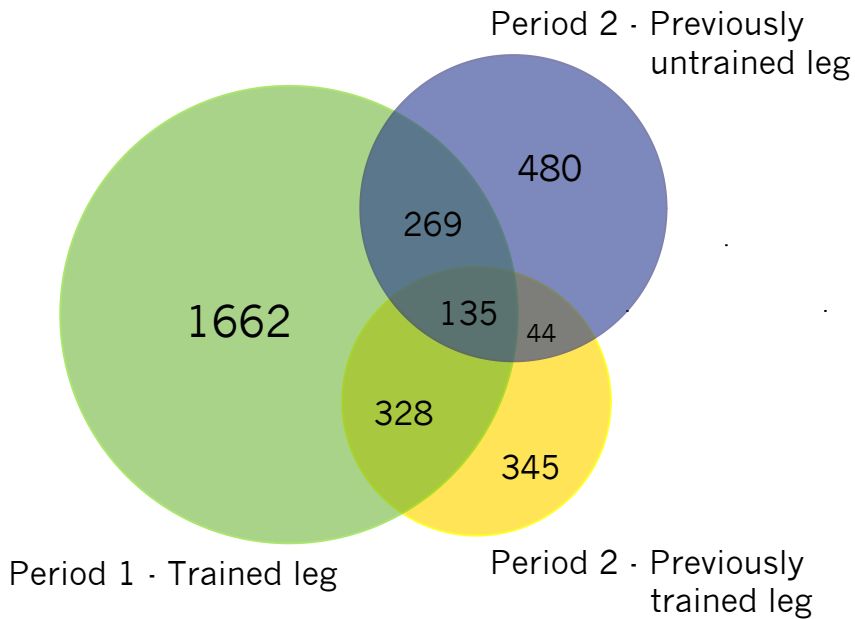

Supplement: S5 Fig — Data is based on loadings from the OPLS analysis shown in Fig 2. (PDF) [file pgen.1006294.s005.pdf]

**a**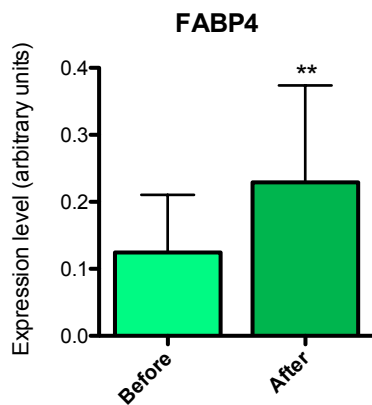**b**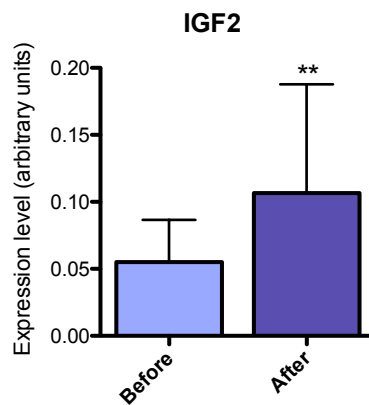**c**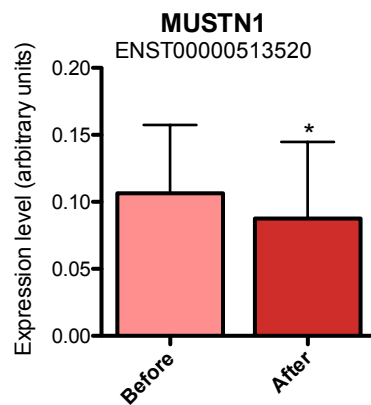**d**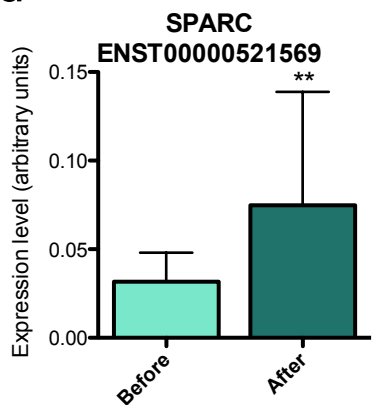**e**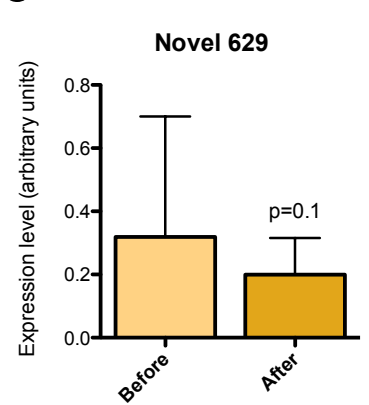**f**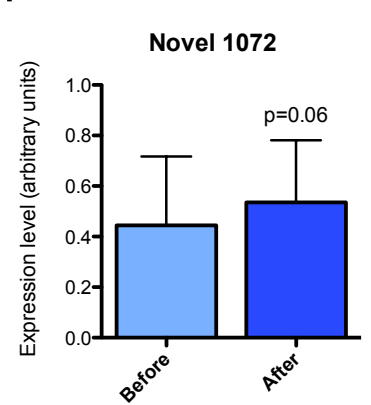**g**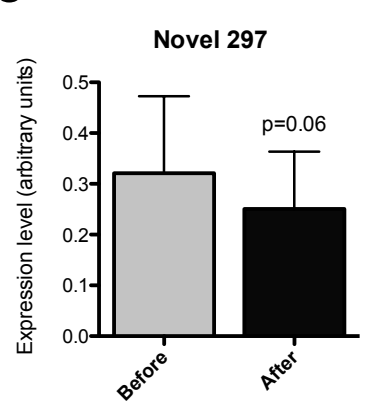**h**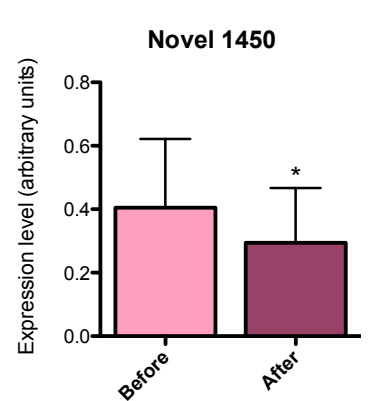**i**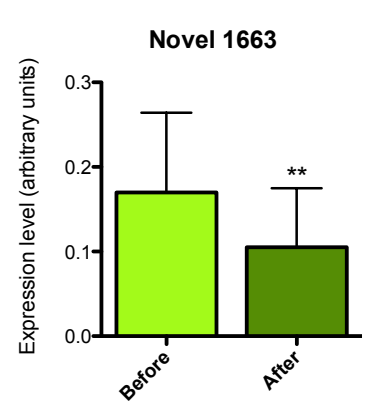

Supplement: S6 Fig — Differentially expressed; known genes a) FABP4 and b) IGF2, known isoforms c) MUSTN1 (ENST00000513520) and d) SPARC (ENST00000521569) (n = 23) and novel transcripts e) Novel 629, f) Novel 1072, g) Novel 297, h) Novel 1450 and i) Novel 1663 before and after training in Period 1 (n = 20). RPS18 was used as an endogenous control for all transcripts. Data is presented as mean ± SD. * p<0.05, ** p<0.01. (PDF) [file pgen.1006294.s006.pdf]
